# Intestinal Immunomodulatory Cells (T Lymphocytes): A Bridge between Gut Microbiota and Diabetes

**DOI:** 10.1155/2018/9830939

**Published:** 2018-03-11

**Authors:** Qingwei Li, Zezheng Gao, Han Wang, Haoran Wu, Yanwen Liu, Yingying Yang, Lin Han, Xinmiao Wang, Linhua Zhao, Xiaolin Tong

**Affiliations:** ^1^Department of Endocrinology, Guang'anmen Hospital of China, Academy of Chinese Medical Sciences, Beijing 100053, China; ^2^Beijing University of Chinese Medicine, Beijing 100029, China; ^3^Shenzhen Hospital, Guangzhou University of Chinese Medicine, Guangzhou 518034, China; ^4^Laboratory of Molecular and Biology, Guang'anmen Hospital of China, Academy of Chinese Medical Sciences, Beijing 100053, China

## Abstract

Diabetes mellitus (DM) is one of the most familiar chronic diseases threatening human health. Recent studies have shown that the development of diabetes is closely related to an imbalance of the gut microbiota. Accordingly, there is increasing interest in how changes in the gut microbiota affect diabetes and its underlying mechanisms. Immunomodulatory cells play important roles in maintaining the normal functioning of the human immune system and in maintaining homeostasis. Intestinal immunomodulatory cells (IICs) are located in the intestinal mucosa and are regarded as an intermediary by which the gut microbiota affects physiological and pathological properties. Diabetes can be regulated by IICs, which act as a bridge linking the gut microbiota and DM. Understanding this bridge role of IICs may clarify the mechanisms by which the gut microbiota contributes to DM. Based on recent research, we summarize this process, thereby providing a basis for further studies of diabetes and other similar immune-related diseases.

## 1. Introduction

The gut microbiota is an important “organ” in the human body; it includes extensive microbes with a wide range of characteristics and has important physiological and pathological functions in the body. Studies of correlations between the gut microbiota and various diseases, such as diabetes, obesity, metabolic syndrome, liver disease, tumors, and functional constipation, have become increasingly popular, and diabetes has been a particular focus of the recent research. It is believed that changes in the gut microbiota significantly influence the onset and development of diabetes mellitus (DM), and increasing studies have evaluated the mechanisms underlying this interaction. Recent studies have shown that the gut microbiota has an impact on the immune system of animals, particularly on intestinal mucosal immunity [1]. Furthermore, extensive immunological and inflammatory responses are involved in the physiological and pathological processes of DM [2, 3]. Thus, we believe that the immune system is a potential link between the gut microbiota and DM.

Intestinal immunomodulatory cells (IICs), a kind of regulatory immune cell in the gastrointestinal system, are believed to be particularly important. IICs control immune responses and reduce inflammation [4]. The immune system is an organic whole, composed of various factors that are coordinated and strictly regulated. Immune effector cells are regulated and controlled by a series of complex, but precise, regulatory networks, and IICs have important roles in maintaining the balance of these networks. Research has shown that the gut microbiota influences IICs to alter immune responses, and by altering the immune responses, IICs influence diabetes development [2, 5]. The position of IICs is critical, and we refer to this key position as the “Bridge.”

We summarize existing literature to explain how the Bridge, that is, the IICs, functions as a link between the gut microbiota and diabetes. In this review, we focus on the intensively studied immunoregulatory cells in the T lymphocyte family, including T helper 1 cells (Th1), T helper 2 cells (Th2), T helper 17 cells (Th17), and regulatory T cells (Treg), to explore the mechanisms by which the gut microbiota affects DM and to clarify the Bridge role of IICs.

### 1.1. Gut Microbiota Closely Related to Diabetes

More than 1000 species of bacteria live in the human intestinal tract. Each species can also be classified into a number of subspecies, totaling over 10^14^, which is almost 10 times greater than the number of cells in the human body. Human microbes weigh approximately 1.275 kg in total, of which those in the gastrointestinal tract account for about 1 kg. These bacteria constitute the intestinal microecosystem [6, 7]. An imbalance of the intestinal microecosystem can cause various diseases, such as functional constipation, Crohn's disease, ulcerative colitis, cirrhosis, fatty liver, senile dementia, and diabetes [8–10]. There are many kinds of intestinal microflora, of which 80% to 90% of taxa are Firmicutes and Bacteroidetes, followed by Actinobacteria and Proteobacteria [11].

Diabetes is a clinical syndrome characterized as a glucose metabolism disorder and is caused by a combination of genetic and environmental factors. Insulin deficiency and insulin dysfunction induce disorders of sugar, fat, protein, water, and electrolyte metabolism, alone or in combination, and chronic hyperglycemia is the main clinical feature [12]. As a multifactorial disease, diabetes requires multivariate treatments for a variety of risk factors. In recent years, the microorganisms in the intestine, also known as the gut microbiota, have been regarded as the main component of the human internal environment and one of the primary environmental factors that determines the improvement or deterioration of DM [13]. Studies have found that type 2 diabetes mellitus (T2DM) is closely related to human nutritional metabolism, which is also substantially influenced by the gut microbiota; thus, we hypothesized that diabetes and the gut microbiota are inextricably linked [14], and this prediction is supported by increasing studies. Further in-depth analyses may identify important indicators and targets for future personalized treatments and methods for the prevention of diabetes.

In early studies of nonobese diabetic (NOD) mice with congenital hypoglycemia, diabetes did not develop in normal conditions, but when mice were kept in sterile environments, they developed severe diabetes, and this was attributed to a lack of beneficial gut microbes [15]. In another study, compared with nondiabetic patients, Firmicutes and Clostridium species in the gut microbiota of patients with diabetes were significantly reduced, and the Bacteroides/Firmicutes ratio and *Pseudobacillus/Escherichia coli* ratio were positively correlated with blood glucose levels, without any relationship to weight. Moreover, *β*-Proteobacteria were significantly enriched and were positively related to blood glucose levels in patients with diabetes [16]. In another study, Cani et al. [3] showed that the combined use of antibiotics in obese (ob/ob) mice fed a high-fat diet resulted in an obvious alteration of the gut microbiota composition, thereby reducing the endotoxin, blood glucose, and glucose tolerance levels in the animal model, indicating that the regulation of the gut microbiota may be beneficial for improving diabetes.

In patients with type 1 diabetes mellitus (T1DM), the composition of the gut microbiota exhibits substantial changes; its alpha diversity is decreased by about 25%, indicating that the cause of T1DM is related not only to susceptibility genes but also to the internal intestinal environment [17]. In another study of the gut microbiota of sixteen 6–8-year-old Caucasian children with T1DM, compared with those of the control group, the number of *Enterobacter cloacae*, *Bacteroides*, and *Veillonella* increased significantly, the number of *Actinomyces*, *Firmicutes*, *Bifidobacterium*, and *Lactobacillus* species decreased, and the ratio of *Bacteroides* to *Firmicutes* increased [18]. Similar changes were observed in patients with type 2 diabetes. Larsen et al. found an obvious increase in *β*-Proteobacteria in patients with T2DM, with reductions in *Bifidobacterium*, *Firmicutes*, and *Clostridium* species; furthermore, the ratios of *Bacteroides/Firmicutes* and *Brevibacterium/Clostridium sphaeroides* were positively correlated with blood glucose levels, demonstrating that changes in the gut microbiota were closely related to the decrease in glucose tolerance [10]. Qin et al. studied fecal samples obtained from 342 Chinese patients with T2DM by intestinal genome sequencing and showed that the gut microbiota of patients with diabetes was changed and some butyric acid-producing bacteria were reduced, but pathogenic bacteria increased in frequency [12].

Additional studies have shown that the gut microbiota is closely linked to the development of diabetes and the underlying mechanism has received increasing interest. The inflammation hypothesis and energy storage hypothesis describe two major mechanisms [19–21]. Despite extensive research, these studies did not examine a particularly wide array of mechanisms and the mechanism is highly complex; accordingly, it is urgent to determine the key mediators that allow the gut microbiota to influence DM. Based on a literature review, we found that intestinal immunoregulatory cells (IICs) are the most common intermediate identified in most of the proposed mechanistic pathways. IICs are immune regulatory cells located in the human intestine, including T lymphocytes, innate lymphoid cells, macrophages, dendritic cells, and mesenchymal stem cells [22]. Among these, T lymphocytes could be classified from a functional point of view into various lineages, including Th1, Th2, Th17, and Treg cells [23]. Here, we define the IIC Bridge position and clarify the Bridge in detail, focusing on Th1, Th2, Th17, and Treg cells, which are the most extensively studied cell subsets. We also demonstrate that the Bridge concept clarifies the mechanism by which the gut microbiota affects DM and provides new ideas and targets for future research, clinical treatments, drug development, and so on.

### 1.2. Effects of the Gut Microbiota and Its Metabolites on Intestinal Immunomodulatory Cells

#### 1.2.1. Effects of the Gut Microbiota and Its Metabolites on TH1/TH2 Cells

A healthy balance of Th1/Th2 cells is essential for immune regulation. IL-12 (p35-p40) and IL-4 are the central cytokines that control the differentiation of Th1 and Th2 cells. These two cytokines induce the production of their respective T cell subsets, while suppressing the production of opposite subsets. IL-12 can promote interferon-*γ* (IFN-γ) and T-bet secretion by T cells and natural killer cells, and these two transcription factors induce the differentiation of Th1 cells via the signal transducer and activator of transcription 4 (STAT4) pathway. IL-4 promotes the secretion of Th2 cytokines by upregulating the expression of GATA binding protein 3 (GATA3) via the activation of the STAT6 pathway [24]. A large number of experiments have shown that the gut microbiota and its metabolites have an effect on the balance of Th1/Th2 cells in the intestinal tract. An experimental study indicated that the gram-negative anaerobic bacterium *Bacteroides fragilis* and its product polysaccharide A could promote the expression of proinflammatory cytokines (IL-12 and p40) and nitrogen oxide in antigen-presenting cells by Toll-like receptor (TLR), thereby activating NF-*κ*B translocation and upregulating IFN-*γ* levels in the body. Under such conditions, the differentiation of Th1 cells was induced by the activation of the STAT4 pathway and major histocompatibility complex II (MHC II) expression [25, 26]. Pam3 of gram-positive bacteria can combine with TLR2 as a ligand and activate IFN-*γ* production via the nuclear factor kappa B (NF-*κ*B) and mitogen-activated protein kinase (MAPK) pathways, including JNK, p38, and ERK. The upregulation of IFN-*γ* induced the differentiation of Th1 cells [27]. The induction of the gut microbiota is related not only to the differentiation of Th1 cells but also to that of Th2 cells; Wu and colleagues found that commensal A4 bacteria belonging to the Lachnospiraceae family, which produce an immunodominant microbiota CBir1 antigen, inhibit Th2 cell development and CBir1 could inhibit Th2 cell differentiation by inducing TGF-*β* production by dendritic cells [28]. In addition, recent research has indicated that yeast *β*-glucan, a polysaccharide, can promote the differentiation and secretory activity of Th2 cells by upregulating the expression of *GATA3* mRNA. Additionally, yeast *β*-glucan upregulates various additional anti-inflammatory cytokines, for example, IL-4, IL-5, IL-15, and IL-33, and downregulates proinflammatory cytokines, including IL-6, TNF-*α*, IL-1*β*, and IL-18, as well as some acute phase proteins, like chemokine C-C motif receptor 2 (CCR2), serum amyloid A3 (SAA3), and orosomucoid 2 (Orm2) [29].

#### 1.2.2. Effects of the Gut Microbiota and Its Metabolites on Th17 Cells

Th17 cells, also known as inflammatory helper T cells, are derived from natural T cell precursors. They have independent mechanisms for differentiation and developmental regulation. Th17 cells mainly secrete IL-17, but not IFN-*γ* and IL-4, and the retinoid-related orphan receptor-*γ*t (ROR-*γ*t) is the specific transcriptional regulator of Th17 cells [30]. Recent studies have found that IL-23, TGF-*β*, and IL-6 could promote the differentiation and expression of Th17 cells; conversely, Th17 cell expression could be inhibited by IL-12, IFN-*γ*, IL-4, T-bet, and Socs3. Some research has shown that segmented filamentous bacteria (SFB) located in the ileum could induce the differentiation of Th17 cells; however, the induction of SFB is unrelated to TLR, NOD-RIP2, or ATP signaling pathways based on analyses in a model of MyD88/TRIF double-deficiency and RIP-2 mutant mice [31, 32]. In another experiment, Ivanov and colleagues found that the main mechanism by which SFB induced the differentiation of Th17 cells was the upregulation of serum amyloid A (SAA) levels [33]. Goto et al. found that SFB antigen presentation by dendritic cells via a major histocompatibility complex (MHC) II-dependent pathway is essential for the induction of Th17 cells, although presentation by group 3 innate lymphoid cells (ILC3) negatively regulates Th17 cell differentiation [34]. Analyses of high-fat diet-induced ROR-*γ*t-deficient and wild-type mice showed that the abundances of Porphyromonadaceae, Peptostreptococcaceae, Comamonadaceae, and Bacteroidaceae were correlated with the expression of ileum IL17 cells and *ROR-γt* mRNA [35].

#### 1.2.3. Effects of the Gut Microbiota and Its Metabolites on Treg Cells

Treg cells play an important role in immunoregulation; according to surface markers, the secreted cytokines, and mechanism of action, Treg cells can be divided into different subtypes. Recent studies of Treg cells have focused on CD4^+^CD25^+^ Treg cells. The differentiation and function of CD4^+^CD25^+^ Treg cells are regulated by the transcription factor Foxp3, antigen-presenting cells, and the cytokines IL-10, IL-12, and TGF-*β*. Additionally, several experiments have indicated that the gut microbiota induces the differentiation and function of Treg cells. *Bacteroides fragilis* and its metabolite polysaccharide A mediate the conversion of CD4^+^ T cells into Foxp3^+^ Treg cells, which produce IL-10 by activating the TLR2 signaling pathway [36]. Clusters IV and XIVa of the genus *Clostridium* promote the expression of Foxp3^+^ Tregs. After the oral inoculation of *Clostridium* in mice, transforming growth factor-*β* (TGF-*β*) levels and Foxp3^+^ Tregs increased, and systemic immunoglobulin E (IgE) responses were inhibited [33]. Tang et al. found that *Lactobacillus murinus* is able to promote the expression of TGF-*β* and IL-10, activating the transcription factor Foxp3 and increasing Treg and Th17 cells in the colon [37]. In addition, the intestinal flora can induce other immune cells by affecting the differentiation of Treg cells. Early in 2009, studies showed that Tregs could express high levels of ROR-*γ*t [38]. SFB and *Lactobacillus casei* BL23 could induce the expression of ROR-*γ*t on Treg cells, and increased ROR-*γ*t^+^ Treg cells induced the differentiation and function of Th17 cells [39, 40]. Geuking et al. found that the altered Schaedler flora, a collection of eight benign intestinal symbiotic microbiota, promoted the production of Treg cells but diminished Th1 and Th17 cell responses [41].

In addition to the gut microbiota, metabolites, especially short-chain fatty acids (SCFA), also affect the differentiation and function of Treg cells. Smith et al. found that SCFAs promoted the differentiation of Treg cells by activating G-protein-coupled free fatty acid receptor 43 (GPR43) [42]. Atarashi and colleagues verified that SCFAs induce the expression of Treg cells by stimulating epithelial cells to produce TGF-*β* [43]. Gpr109A (encoded by Niacr1) is a receptor for butyrate and niacin in the colon. Butyrate is able to induce the differentiation of Treg cells and IL 10-producing T cells by binding to Gpr109A. Furthermore, Gpr109a was essential for the butyrate-mediated induction of IL-18 in the colonic epithelium. Animal experimental studies have shown that colitis and colon cancer develop easily in Niacr1^−/−^ mice, which is a model of a lack of GPR109A expression [44]. The SCFA butyrate also induces an increase in ROR-*γ*t^+^ Treg cells, dependent on dendritic cells and MHC II, and thereby affects the differentiation and function of other immune cells [39].

### 1.3. Effects of Intestinal Immunomodulatory Cells (T Lymphocytes) on Diabetes

#### 1.3.1. Effects of Th1/Th2 Cells and Their Cytokines on Diabetes

The differentiation of early CD4^+^ T cells is based on a simple dichotomy between interferon- (IFN-) *γ*-dominated Th1 cell responses and IL-4-dominated Th2 cell responses. Th1 cells can be induced by IL-12 and are key elements for macrophage activation and the clearance of intracellular pathogens, while Th2 cells defend against helminth infections, which are related to allergic disorders, along with IgE, mast cells, and eosinophils.

Th1 cells mainly produce IFN-*γ*, and IL-12 could lead to the activation of CD8^+^ T cells, thus resulting in the destruction of islet *β* cells. The destruction of *β* cells occurs by the infiltration by T cells and the secretion of cytotoxic factors by Th1 cells [45]. Th1- and Th2-mediated immunities are reciprocally regulated and maintain a balance in immune-mediated diseases [46]. In Th1 cells, IFN-*γ* could activate macrophages to exert cytotoxic activities by the secretion of toxic cytokines [47]. Studies have indicated a direct role of IFN-*γ* in driving the disease process. Sarvetnick et al. found that the expression of IFN-*γ* under the control of the human insulin promoter is sufficient to cause the development of diabetes in mice [48] and, conversely, the blockade of IFN-*γ* in NOD mice could prevent diabetes [49].

IL-12 is another important regulator of Th1 cell differentiation, and it is also the primary immunoregulatory factor secreted by Th1 cells. IL-12 plays a key role in the pathogenesis of diabetes and its complications. Weaver and Nadler found that IL-12 is able to bind to IL-12 receptors on pancreatic islet *β* cells and activate proinflammatory cytokines (IL-1*β*, TNF-*α*, and IFN-*γ*), resulting in the induction of the apoptosis of islet *β* cells via the STAT4 signaling pathway [50]. In addition, IL-12 contributes to the development of complications during type 2 diabetes, and angiogenesis and arteriogenesis were induced in mice with T2DM via an eNOS/Akt/VEGFR2/oxidative stress/inflammation-dependent mechanism [51].

Some Th2 cell cytokines, for example, IL-10, can specifically enhance major histocompatibility complex class II expression, thus promoting peri-insulitis. They could also activate resident immune cells, establishing anti-*β* cell immunity, leading to pancreatic infiltration from other types of cells [52]. Sokolova et al. verified that the serum levels of the Th2 cell cytokines IL-4 and IL-5 were significantly higher in patients with T2DM than those in a healthy control group with a BMI of 18–24.9 kg/m^2^ [53]. Anand et al. detected a mixed Th1/Th2 cytokine profile in patients with T2DM, and they also found higher serum levels of the Th2 cytokines IL-4 and IL-13 in T2DM patients compared with those of the control group [54]. Kang et al. demonstrated that IL-4 is involved in metabolic control, improving insulin sensitivity and glucose tolerance [55]. The pathophysiological and immunological mechanisms linking increased serum levels of IL-4 and the occurrence of T2DM, metabolic control, and insulin resistance are still unclear. IL-4, a major immunoregulatory factor secreted by Th2 cells, plays an important role in protecting pancreatic *β* cells, reducing the inflammation level in insulin target organs and thus alleviating insulin resistance. IL-4 is able to improve insulin sensitivity by upregulating the level of Akt phosphorylation and attenuating the activation of GSK-3b, while IL-4 could regulate fat metabolism and inhibit fat accumulation by affecting the levels of adipokines (adiponectin and leptin) and free fatty acids [56].

IL-10 acts as an immunoregulatory factor secreted by Th2 and Treg cells and plays an important role in regulating autoimmunity, reducing inflammation levels, and alleviating insulin resistance and metabolic disorders. Studies have shown that IL-10 could bind to IL-10 receptor on macrophages, thus inhibiting macrophage activation and the secretion of inflammatory factors by activating the Jak1/STAT3 or Tyk2/STAT3 signaling pathway [57–59]. Glucose transmembrane transport is performed by glucose transporters (GLUTs) on the cell membrane. GLUT4 is widely expressed in various types of cells, such as muscle and adipose tissue cells. Skeletal muscle is an important organ of glucose metabolism, and the aggregation of lipid-mediated inflammatory factors (TNF-*α*, IL-1*β*, and IL-6) is a main cause of insulin resistance. IL-10 binds to IL-10 receptor on the membrane of skeletal muscle cells, alleviating oxidative stress and inflammation, thus promoting glucose metabolism [60].

#### 1.3.2. Effects of Th17/Treg Cells and Their Cytokines on Diabetes

Th17 and Treg cells are two CD4^+^ T helper cells that can be derived from the same naive CD4^+^ T cells depending on the amounts of TGF-*β* and proinflammatory cytokines. Treg cells regulate and control immune tolerance in healthy individuals, while Treg cell dysfunction or a decrease in their amount might result in excessive immune attacks and autoimmune diseases [61]. In some animal models, CD4^+^ CD25^+^ FoxP3^+^ Tregs could stop the destruction of pancreatic islets and protect against autoimmune T1DM [62]. Yuan et al. indicated that the percentage of CD4^+^ CD25^+^ Foxp3^+^ Tregs and levels of related cytokines (mainly IL-10 and TGF-*β*) were precipitously decreased in patients with newly diagnosed T2DM [63]. After 2 weeks of treatment with Tregs, patients exhibit a significant decrease in the requirement of exogenous insulin and a decrease in HbA1c levels; furthermore, an increase in the percentage of Tregs in the peripheral blood was also observed since the day of treatment [64].

Th17 cells mainly secrete the signature cytokine IL-17A (commonly referred to as IL-17); they also produce IL-17F, IL-21, IL-22, and granulocyte monocyte colony-stimulating factor (GM-CSF) and potentially produce TNF and IL-6 [65]. The cytokines produced by Th17 cells have a wide range of effects on many types of cells and could induce the secretion of proinflammatory cytokines and chemokines. However, in some instances, IL-17-driven inflammation does not have a protective effect but is a risk factor for severe immunopathology and autoimmunity [66].

Martin-Orozco et al. evaluated a spontaneous autoimmune diabetes model and showed that IL-17A and IL-17F expressions in islets are related to insulitis in NOD mice. The increased expression of Th17 cells is often accompanied by Th1 cells or IFN-*γ*. Moreover, islet antigen-specific Th17 cells need to be transformed into Th1-like cells to induce diabetes [67]. Arif and colleagues claimed that a feature of T1DM is the autoreaction of IL-17^+^-specific CD4^+^ T cells, and the inhibition of Th17 cells reduced the islet-specific inflammatory T cell infiltration [68]. Honkanen and colleagues clearly demonstrated a Th17-based response in patients with T1DM. The peripheral blood CD4^+^ T cells from children with new onset T1DM secrete higher levels of IL-17 and IL-22 than those from healthy individuals upon polyclonal activation, and there was no increase in IFN-*γ* levels or T-bet expression in patients with T1DM [69]. According to another study, the cAMP-mediated production of macrophage-derived prostaglandin E2 (PGE2) stimulates Th17 cell production of IL-17A and IL-17F via the cAMP-response element binding protein/CREB-regulated transcription coactivator 2 (CREB/CRTC2) pathway [70]. IL-17 binds to IL-17 receptor of islet *β* cells and upregulates the expression of STAT1 and NF-*κ*B in cells, which results in the apoptosis of islet *β* cells [68, 71]. In addition to direct destruction in islet cells, Saxena et al. also found that IL-17 could induce the differentiation of Th1 cells and then cooperate with Th1 cells to mediate autoimmune diseases [72].

Zhang et al. showed that the duration of diabetes was positively related to the proportion of Th17 cells and negatively related to the proportion of Treg cells. Furthermore, they did not detect significant relationships between diabetes duration and Th1 cells or Th2 cells, and they supposed that this parameter may have an impact on the Th17/Treg paradigm, rather than the Th1/Th2 paradigm [73]. The balance between Th17 and Treg cells is a key factor in autoimmune diseases, and an imbalance typically involves an increase in Th17 cells, but a decrease in Treg cells, which leads to a failure of the regulation on ongoing immune reactions. For example, in NOD mice, a Th17/Treg imbalance weakens the ability of Treg cells to suppress self-reactive effector T cell activity and to stop the destruction of pancreatic islets, which may potentially induce or aggravate T1DM [64].

## 2. Conclusions

DM is a common metabolic disorder with a high morbidity and a high risk of severe complications, resulting in substantial damage to multiple organs in humans. Without any effective long-term treatment, patients with diabetes usually have a rather low quality of life, and this situation has prompted studies of diabetes from multiple perspectives. Recently, increasing studies have shown that the gut microbiota participates in the maintenance of human health and the development of diseases and is directly related to the occurrence and development of diabetes. The gut microbiota and related topics have become a focus of recent research and provide an opportunity to make great progress in our understanding of diabetes, with the potential to facilitate mechanistic research, clinical trials, drug development, and improving the efficacy of treatment strategies.

**Figure 1:**
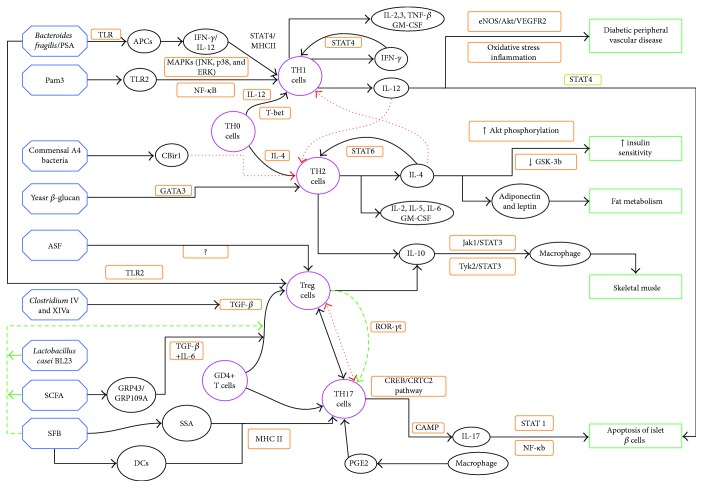
Blue indicates the gut microbiota, purple indicates T lymphocytes, and green indicates different targets of diabetes. Red lines represent inhibitory action. Solid black lines represent a positive effect (promotion). PSA: polysaccharide A; ASF: altered Schaedler flora; SCFA: short-chain fatty acids; SFB: segmented filamentous bacteria; APCS: antigen-presenting cells; TLR: Toll-like receptor; TH0, 1, 2, and 17 cells: T helper 0, 1, 2, and 17 cells; Treg: regulatory T cell; SAA: serum amyloid A; MAPK: mitogen-activated protein kinase; JNK: c-Jun N-terminal kinase; ERK: extracellular signal-regulated kinase; NF-*κ*B: nuclear factor kappa B; STAT4 and 6: signal transducer and activator of transcription 4 and 6; MHC: major histocompatibility complex; eNOS: endothelial nitric oxide synthase; VEGFR2: vascular endothelial growth factor receptor 2; Jak: Janus kinase; Tyk: tyrosine kinase; CREB: cAMP-response element binding protein; CRTC2: CREB-regulated transcription coactivator 2.

Based on a number of studies, we identified a key link between the gut microbiota and diabetes, or a Bridge material, that is, IICs. Many cell types are classified as IICs, and this review focuses on T lymphocytes, which have been evaluated extensively in recent research, to describe the mechanisms by which the gut microbiota affects diabetes. We developed a flow chart to clearly display the complex mechanisms described in this review (Figure 1). As this graph shows, many gut microbiota components regulate the differentiation and function of T lymphocytes via a variety of pathways. T lymphocytes secrete various cytokines, and these play important roles in regulating autoimmunity, protecting islet cells, improving glucose and lipid metabolism, and reducing insulin resistance, thus alleviating diabetes. Interestingly, Th1, Th2, Th17, and Treg cells come from the T lymphocytes, and they are not only able to regulate each other, but they also have antagonistic effects depending on their conditions, consequently alleviating or aggravating the syndrome of diabetes. These findings provide new targets to develop therapies. T lymphocytes are not the only IICs, and further studies are necessary to examine a wider array of cellular mechanisms and disease characteristics.
